# Astrocytic GABA Accumulation in Experimental Temporal Lobe Epilepsy

**DOI:** 10.3389/fneur.2020.614923

**Published:** 2020-12-18

**Authors:** Julia Müller, Aline Timmermann, Lukas Henning, Hendrik Müller, Christian Steinhäuser, Peter Bedner

**Affiliations:** Institute of Cellular Neurosciences, Medical Faculty, University of Bonn, Bonn, Germany

**Keywords:** temporal lobe epilepsy, hippocampal sclerosis, astrocyte, tonic current, GABA

## Abstract

An imbalance of excitation and inhibition has been associated with the pathophysiology of epilepsy. Loss of GABAergic interneurons and/or synaptic inhibition has been shown in various epilepsy models and in human epilepsy. Despite this loss, several studies reported preserved or increased tonic GABA_A_ receptor-mediated currents in epilepsy, raising the question of the source of the inhibitory transmitter. We used the unilateral intracortical kainate mouse model of temporal lobe epilepsy (TLE) with hippocampal sclerosis (HS) to answer this question. In our model we observed profound loss of interneurons in the sclerotic hippocampal CA1 region and dentate gyrus already 5 days after epilepsy induction. Consistent with the literature, the absence of interneurons caused no reduction of tonic inhibition of CA1 pyramidal neurons. In dentate granule cells the inhibitory currents were even increased in epileptic tissue. Intriguingly, immunostaining of brain sections from epileptic mice with antibodies against GABA revealed strong and progressive accumulation of the neurotransmitter in reactive astrocytes. Pharmacological inhibition of the astrocytic GABA transporter GAT3 did not affect tonic inhibition in the sclerotic hippocampus, suggesting that this transporter is not responsible for astrocytic GABA accumulation or release. Immunostaining further indicated that both decarboxylation of glutamate and putrescine degradation accounted for the increased GABA levels in reactive astrocytes. Together, our data provide evidence that the preserved tonic inhibitory currents in the epileptic brain are mediated by GABA overproduction and release from astrocytes. A deeper understanding of the underlying mechanisms may lead to new strategies for antiepileptic drug therapy.

## Highlights

- Despite massive loss of interneurons, tonic GABA_A_ receptor-mediated currents are preserved in the sclerotic hippocampal CA1 region and increased in the dentate gyrus.
- Reactive astrocytes in the sclerotic mouse hippocampus display pronounced GABA accumulation.
- Both decarboxylation of glutamate and putrescine degradation may underlie astrocytic GABA accumulation.

## Introduction

Epilepsy is a disorder of the brain characterized by recurrent unprovoked seizures that affects 1–2% of the population worldwide (1). Temporal lobe epilepsy (TLE), the most frequent and severe form of focal epilepsy in adults is particularly difficult to control with antiepileptic therapies. Despite the availability of third-generation antiepileptic drugs (AEDs) a high proportion of TLE patients do not respond adequately to medication (2, 3). The main goal of epilepsy research is, therefore, to identify new therapeutic targets and strategies for the development of more effective and better tolerated AEDs. The most common pathologic finding in patients with TLE is hippocampal sclerosis (HS), histologically characterized by segmental loss of principal pyramidal neurons, synaptic reorganization and reactive astrogliosis in the hippocampus (4). In addition, loss of hippocampal GABAergic interneurons has been described in human TLE (5–9) and in many different animal models (10–13). The consequential shift in the excitation-inhibition balance toward excitation has been hypothesized to represent the primary cause of seizure activity in TLE (11, 14–17). However, this hypothesis has difficulty explaining the fact that epileptic seizures are intermittent and relatively rare events even in patients and animals with severe epilepsy, pointing to the existence of a compensatory mechanism that restores the excitation-inhibition balance to a large extent (18–20). Most recent work suggested that the compensation is accomplished by channel-mediated tonic GABA release from reactive astrocytes (21), a mechanism that was proposed to be relevant also in other neurological disorders, such as Alzheimer's disease, Parkinson's disease or stroke (22–25). According to this scenario, reactive astrocytes aberrantly overproduce and release GABA, which in turn inhibits neuronal excitability and network activity through activation of high affinity, slowly desensitizing extrasynaptic GABA_A_ receptors (GABA_A_Rs) (20). In line with this view, evidence from animal models indicate that despite the loss of synaptic inhibition, tonic GABA_A_R-mediated currents (often termed “tonic inhibition”) are preserved or even increased in focal epilepsy (18, 20, 21). Hence, it was suggested that reactive astrocytes suppress network excitability and prevent seizure generation through tonic GABA release, a pathway that could provide an attractive target for the development of new AEDs.

To gain further insight into this highly relevant topic, in the present study we used immunohistochemical and electrophysiological methods to unravel the relationship between the extent of interneuronal loss, tonic inhibition and astrocytic GABA content in the hippocampal CA1 region and dentate gyrus during the early chronic phase of epileptogenesis in the unilateral intracortical kainate mouse model of TLE-HS. The results further support the hypothesis that the preserved tonic inhibition in TLE-HS is mediated by ambient GABA released from reactive astrocytes.

## Materials and Methods

### Animals

Maintenance and handling of animals was according to the local government regulations. Experiments were approved by the North Rhine–Westphalia State Agency for Nature, Environment and Consumer Protection (approval numbers 84-02.04.2012.A212 and 84-02.04.2015.A393). All measures were taken to minimize the number of animals used. Mice were kept under standard housing conditions (12/12 h dark–light cycle, food, and water *ad libitum*). Male FVB (Charles River, Sulzfeld, Germany) or transgenic mice with human GFAP (hGFAP) promoter-controlled expression of EGFP [hGFAP/EGFP (26)] aged 90–120 days were used for the experiments.

### Unilateral Intracortical Kainate Injections

We used the TLE animal model previously established (27, 28). Briefly, mice were anesthetized with a mixture of medetomidine (Cepetor, CP-Pharma, Burgdorf, Germany, 0.3 mg/kg, i.p.) and ketamine (Ketamidor, WDT, Garbsen, Germany, 40 mg/kg, i.p.) and placed in a stereotaxic frame equipped with a manual microinjection unit (TSE Systems GmbH, Bad Homburg, Germany). A total volume of 70 nl of a 20 mM solution of kainic acid (Tocris, Bristol, UK) in 0.9% sterile NaCl were stereotactically injected into the neocortex just above the right dorsal hippocampus. The stereotactic coordinates were 2 mm posterior to bregma, 1.5 mm from midline and 1.7 mm from the skull surface. Sham control mice received injections of 70 nl saline under the same conditions. For the analysis of tonic GABA_A_R currents untreated mice served as controls. After injection, the scalp incision was sutured and anesthesia stopped with atipamezol (Antisedan, Orion Pharma, Hamburg, Germany, 300 mg/kg, i.p.). To reduce pain, mice were subsequently injected for 3 days with carprofen (Rimadyl, Pfizer, Karlsruhe, Germany). Furthermore, 0.25% Enrofloxacin (Baytril, Bayer, Leverkusen, Germany) was administered via drinking water to reduce the risk of infection. Brains of the mice were perfusion fixed with 4% PFA followed by overnight fixation in 4% PFA.

### Immunhistochemistry

#### Tissue Preparation

Adult animals were deeply anaesthesised by intraperitoneal (i.p.) injection with 100–120 μl of a solution containing 80 mg/kg ketamine hydrochloride (WDT) and 1.2 mg/kg xylazine hydrochloride (Sigma-Aldrich, Darmstadt, Germany). After testing the hind paw reflexes, transcardial perfusion was applied with ice-cold PBS (30 ml) followed by ice-cold PFA (30 ml, 4%). The brain was removed and an additional fixation with 4% PFA overnight was performed. Tissue was stored in PBS at 4°C until sectioning.

#### Staining

Slices from PFA-perfused animals were cut into 40 μm thickness with a vibratome. Each slice was transferred into a well of a 24-well plate and able to freely move during the whole staining procedure. Only dorsal hippocampal slices close to the injection site (1.8–2.2 mm from bregma) were used for staining. To avoid unspecific binding of antibodies, all slices were incubated in blocking solution for 1.5–2 h at room temperature (RT), containing PBS 0.5–1% TritonX-100 for cell membrane permeabilisation and normal goat serum (NGS, 10%). Primary antibodies were diluted in PBS, 0.1% TritonX-100 and 5% NGS and the slices were incubated with overnight shaking at 4°C (except for GABA staining where slices were incubated for 48 h at RT). The following primary antibodies were used: mouse-anti-S100b (1:200, Abcam, Cambridge, UK), rabbit-anti-GABA (1:2000; ImmunoStar, Hudson, WI, USA), rabbit-anti-GFAP (1:500, DAKO, Hamburg, Germany), mouse-anti-PARV (1:1000, Millipore, Darmstadt, Germany) rabbit-anti-GAD65+67 (1:1000, Sigma-Aldrich, Steinheim, Germany), rabbit-anti-MAO-B (1:500, Sigma-Aldrich, Steinheim, Germany). On the following day every slice was washed three times with PBS for 10 min each, followed by incubation with secondary antibodies conjugated with Alexa Fluor 488, Alexa Fluor 594 or Alexa Fluor 647 (Invitrogen, dilution 1:500 each) in PBS with 2% NGS and 0.1% Triton X-100 for 2 h at RT. After washing them again three times with PBS for 10 min, nuclear staining with Hoechst (1:200, diluted in dH_2_O) was performed (10 min, RT). A final washing step was performed and slices were mounted with Aquapolymount (Polysciences, Heidelberg, Germany) on objective slides and covered with cover slips. Before confocal imaging, slides were stored at 4°C overnight.

### Confocal Microscopy

Slides were examined using a confocal laser scanning microscope (SP8, Leica, Hamburg, Germany) in either standard or photon counting mode (8 bit) using 20 or 63 × objectives. Image resolution was 1,024 × 1,024 pixels taken at a speed of 400 Hz, with a pinhole of 1.2 or 1 au and zoom 0.75 or 1. For detection of Hoechst, a photomultiplier was used, whereas for all other staining hybrid detectors were acquired with the same laser settings. For the 63 × immersion objective a motor correction was performed to improve resolution, depth of penetration and signal strength. Z-stacks were taken as 2 μm thick planes.

### Quantification of Staining

Immunohistochemical data were quantified using the software Imaris 8.0 (Bitplane, Zürich, Switzerland). 3D images (246 × 246 × 40 μm^3^ in size, taken in the CA1 and DG area directly below the injection site) were loaded into the software and co-localization between GFAP and GABA-positive voxels was determined based on an automatic threshold algorithm implemented in the ImarisColoc tool (29), creating a separate fluorescence intensity channel containing only co-localized GFAP- and GABA-positive voxels. The thresholding procedure was applied equally to all images analyzed. Co-localized voxels were subsequently reconstructed creating 3D surface representations (isosurfaces) of the GFAP-positive voxels containing GABA. Additionally, GFAP-negative surfaces with roundish cell bodies were identified as neurons, as confirmed through double staining with antibodies for NeuN and GABA (data not shown). After 3D surface reconstruction, GABA concentration was determined by quantifying total fluorescence intensity of GABA within 3D surfaces representing GFAP/GABA-positive cells. For statistical analysis image data from five animals per group were considered. The number of GABA- and PARV-positive interneurons was counted manually in an area of 246 × 246 × 40 μm^3^ or 582 × 582 × 40 μm^3^, respectively, in the CA1 and DG area below the injection track on the ipsilateral side and at the same position on the contralateral side. Expression of MAO-B and GAD in astrocytes was quantified based on the same procedure described above for quantification of GABA.

### Analysis of Tonic GABA_A_R Currents

Neuronal tonic GABA_A_R currents were measured in coronal brain slices of 200 μm thickness. For kainate injected mice, slices were obtained 2 weeks post-injection and neurons in both, contra and ipsilateral side, were recorded. Untreated mice were used as controls. Slices were prepared as mentioned before and allowed to recover for at least 1 h prior to the experiments. Patch-Clamp recordings were performed at RT at an upright microscope (Axioskop FS2, Zeiss, Jena, Germany) equipped with a CCD camera (VX45, Optronis, Kehl, Germany), infrared-DIC optics (Eclipse E600 FN; Nikon, Japan) and epifluorescence (Polychrome II, Till Photonics, Martinsried, Germany). Slices were constantly perfused with aCSF containing (in mM): 126 NaCl, 3 KCl, 2 MgSO_4_, 2 CaCl_2_, 10 glucose, 1.25 NaH_2_PO_4_, and 26 NaHCO_3_ (pH 7.4, 305–315 mOsm). Whole-cell recordings were obtained from granule cells located in dentate gyrus and from CA1 pyramidal cell neurons located close to the CA2 region. The holding potential was −70 mV. Patch pipettes with a resistance of 3–5 MΩ were filled with an internal solution (in mM): 130 CsCl, 2 MgCl_2_, 0.5 CaCl_2_, 10 HEPES, 5 BAPTA, 3 Na2-ATP and 5 QX-314 (blocker of voltage gated Na^+^ currents) (pH 7.3, 278–285 mOsmol). To isolate tonic GABA_A_R currents, focal pressure applications were performed with an Octaflow system (ALA Scientific Instruments, Farmingdale, NY, USA). The different channels of the application system contained either aCSF (initial control) or a blocker cocktail containing D-AP5 (10 μM, Abcam, Cambridge, UK), NBQX (5 μM, Tocris) and CGP52432 (5 μM, Abcam) w/o bicuculline (20 μM, Tocris), duration of application always 30–50 s. Another channel was loaded with the cocktail plus SNAP5114 (100 μM, Tocris, application for 300 s), and finally the SNAP-containing blocker cocktail was supplemented with bicuculline (application for 30 s). The shift in baseline (i.e., tonic inward) current upon bicuculline application (with or without SNAP) was analyzed with Igor Pro 5.03 software (WaveMetrics, Lake Oswego, OR, USA) and Origin 9.1 (OriginLab Corporation, Northampton, MA, USA). Signals were obtained with an EPC800 amplifier (HEKA Electronic, Lambrecht, Germany) and processed by a differential amplifier (DPA-2FS; npi electronic, Tamm, Germany). Spontaneous inhibitory post-synaptic currents (sIPSCs) were analyzed by the software pClamp (Molecular devices, San José, USA). Individual sIPSCs were identified by a template search, representing sIPSCs in their shape and kinetics. The template was generated from the average of several sIPSCs and kept constant for all experiments. The peak amplitude of each identified sIPSC was measured and the mean amplitude of all recorded cells calculated. Signals were digitized with an ITC 16 D/A converter (HEKA) and displayed with TIDA software (HEKA). Signals were filtered at 1 kHz and sampled at 20 kHz.

### Statistics

Statistical analyses were performed using Origin (OriginLab, version 9, US) and R software [R Core Team 2020, version 4.0.2, Austria (30)]. Data are displayed as mean ± SD. To test whether the data follow a Gaussian distribution both histograms as well as Q-Q plots, which represent the relationship between the percentiles of the theoretical and empirical distributions, were visually inspected. In addition the data were statistically tested for normality (Shapiro-Wilk test). In case of a deviation from normality data were transformed according to Tukey's ladder of powers prior to conducting statistical analysis. For comparison of two groups a Student's *t*-test was used. More than two groups were compared with one-way analysis of variance (ANOVA) with *post-hoc* Tukey test. For multifactorial data stratified two-way ANOVA was conducted. Spearman's rank correlation coefficient was calculated to assess the correlation between GFAP and astrocytic GABA immunoreactivity. Differences between means were considered as being significant at *p* ≤ 0.05. Box plot data represent median (line) and quartiles (25 and 75%; box), whiskers extend to the highest and lowest values within 1.5 times interquartile range.

## Results

### Loss of Hippocampal Interneurons in the Intracortical Kainate Model of TLE

**Figure 1:**
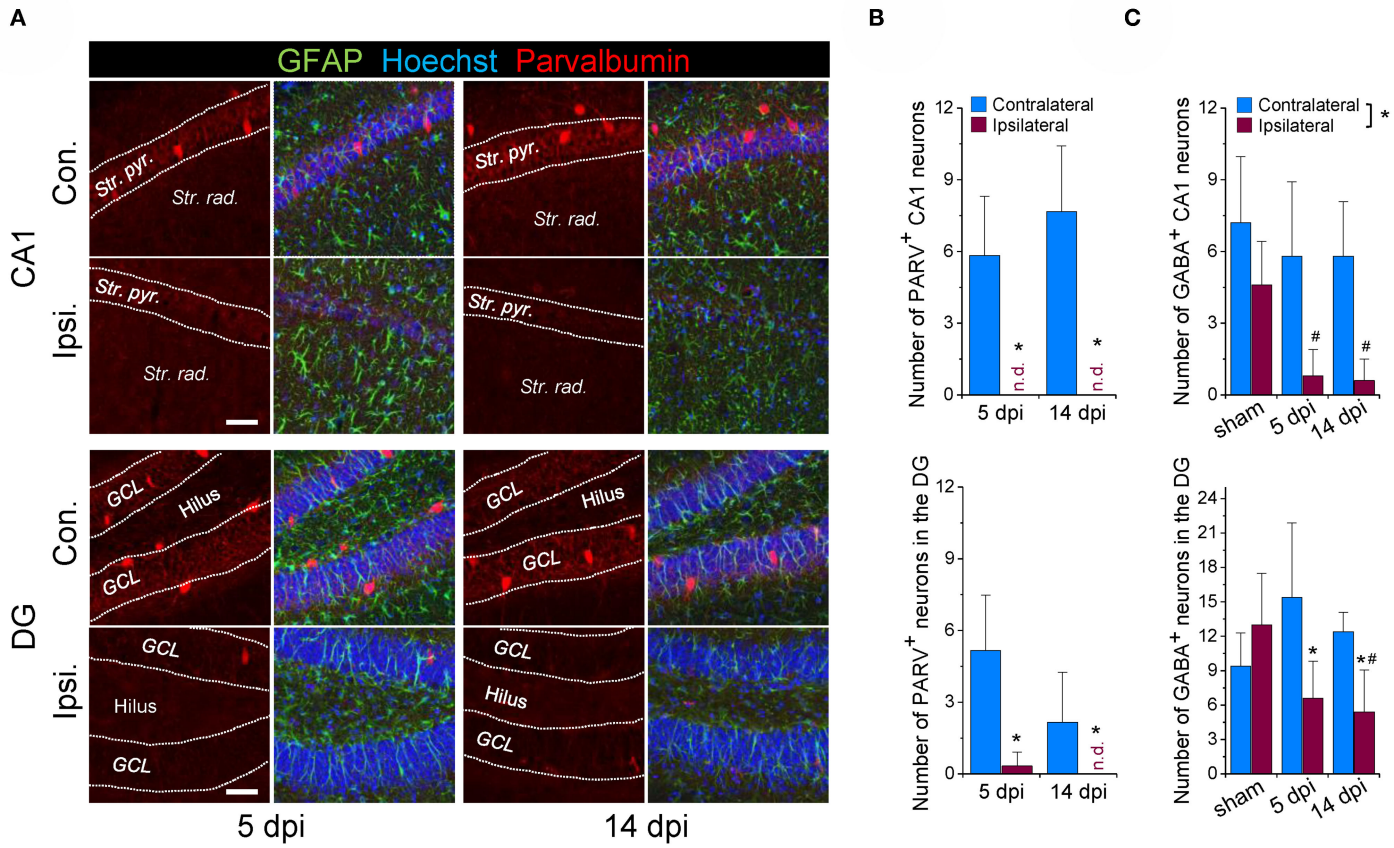
Loss of GABAergic interneurons during the early phase of kainate-induced epileptogenesis. **(A)** Representative confocal images of parvalbumin (PARV) immunoreactivity in the hippocampal CA1 region and dentate gyrus (DG) of mice injected with kainate 5 days and 14 days before. **(B)** Quantification of the number of parvalbumin-positive neurons in an area of 582 × 582 × 40 μm within the hippocampal CA1 and DG region below the injection site. *N* = 6 slices from 3 animals for each time point and area. **(C)** Quantification of the number of GABA-positive neurons (GABA staining shown in **Figure 3**) in the ipsi- and contralateral hippocampus of sham and kainate injected animals. Cells were counted in an area of 246 × 246 × 40 μm within the hippocampal CA1 and DG region below the injection site. *N* = 5 slices from five animals (GABA) for each time point, area and condition. Str. pyr. = Stratum pyramidale, Str. rad. = Stratum radiatum, dpi = days post-injection, n.d. = not detected. ^*^ipsi- vs. contralateral significantly different, ^#^significantly different from sham (*p* < 0.05, stratified two-way ANOVA followed by Tukey's test). Scale bar = 50 μm.

Loss of GABAergic interneurons, especially those containing parvalbumin (PARV), has been documented in human epilepsy and in several different experimental models of the disease, suggesting that it is critically involved in epileptogenesis. We have previously shown that the unilateral intra*cortical* kainate injection model reliably mimics key morphological and functional features of chronic human TLE-HS (27). However, whether the model also reproduces the reported loss of interneurons has not been investigated so far. We used immunohistochemical staining with antibodies against PARV and GABA to tackle this question. Experiments were performed 5 and 14 days after kainate injection (dpi), time points that represent the onset and the early stage of chronic seizure activity in this model. On the contralateral (non-injected) side, abundant PARV-positive cells were detected in the hippocampal CA1 region and DG at both time points. In contrast, cells displaying PARV-immunoreactivity were virtually absent ipsilaterally (Figures 1A,B). Since only a subset of interneurons expresses PARV, we used next anti-GABA antibody to label all types of GABAergic interneurons. Co-staining with the astrocyte marker GFAP revealed an almost complete (~90%) loss of GABA-positive/GFAP-negative cells in the sclerotic hippocampal CA1 region and a substantial reduction (>50%) in the DG at both time points investigated (Figure 1C, representative immune staining are shown in **Figures 3A,B**). The number of contralateral GABAergic interneurons was not different from sham injected controls (Figure 1C).

Together, these findings are consistent with other work demonstrating loss of interneurons in epilepsy, and indicate that this pathological process represents a very early event during epileptogenesis.

### Tonic GABA_A_R-Mediated Currents Are Maintained in CA1 Pyramidal Cells and Increased in Dentate Granule Cells in Experimental TLE-HS

**Figure 2:**
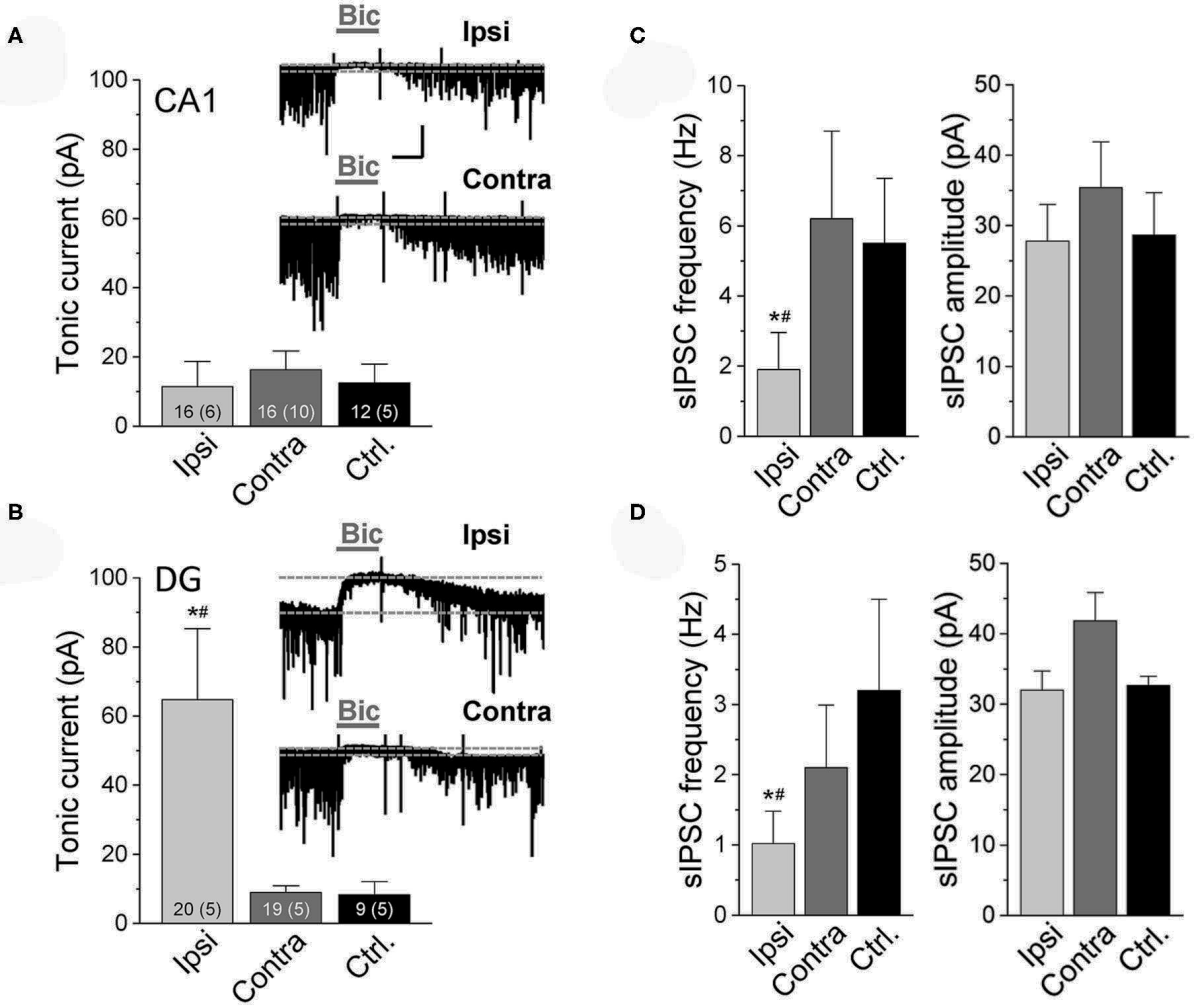
Tonic GABA_A_R currents recorded in CA1 pyramidal neurons and dentate granule cells of epileptic mice. Currents were measured in the ipsilateral (Ipsi) and contralateral (Contra) hippocampus of kainate-treated mice and in the hippocampus of untreated control mice (Ctrl.) 2 weeks after kainate injection. Whole-cell recordings were made with a CsCl-based internal solution, holding potential −70 mV. Tonic current amplitudes represent the shift in baseline current produced by bicuculline (Bic, 20 μM). **(A)** Representative traces of GABA_A_R-mediated currents and their quantification in CA1 pyramidal neurons and **(B)** dentate granule cells. Numbers of experiments and mice (in parentheses) are given in bars. **(C,D)** Frequency and amplitudes of spontaneous inhibitory post-synaptic currents (sIPSCs) recorded before application and after washout of bicuculline in the CA1 and DG, respectively. Numbers of experiments and mice correspond to those in **(A)** and **(B)**. Calibration bars for original traces in **(A)** indicate 20 s and 50 pA and also apply to **(B)**. Error bars represent SD. *significantly different from the contralateral side, #significantly different from controls (*p* < 0.05, one-way ANOVA followed by Tukey's test).

The reduced number of GABAergic interneurons in our experimental model prompted us to examine the magnitude of tonic inhibition in hippocampal neurons. As vesicular release from interneurons has been suggested to be the main source of ambient GABA responsible for tonic inhibition (31), one would expect a reduction in the amplitude of these currents in the sclerotic hippocampus. To test this assumption, we performed electrophysiological recordings from CA1 pyramidal neurons and dentate granule cells 14 dpi. Whole-cell patch-clamp recordings were made using a CsCl-based pipette solution at a holding potential of −70 mV in the presence of the ionotropic glutamate receptor antagonists D-AP5 and NBQX and the GABA_B_ receptor antagonist CGP52432. Tonic current amplitude was calculated as the difference in holding current before and after bicuculline (20 μM) application. Interestingly, tonic inhibition on the ipsi- vs. contralateral sides in kainate injected mice and vs. untreated control animals were not different in CA1 pyramidal neurons (ipsi: 11.4 ± 7.3 pA; contra: 16.3 ± 5.4 pA; control: 12.5 ± 5.4 pA, Figure 2A). In dentate granule cells the amplitudes of tonic currents were even higher at the ipsi- vs. contralateral sides and controls (ipsi: 64.7 ± 20.6 pA; contra: 8.98 ± 1.9 pA; control: 8.3 ± 3.8 pA, Figure 2B). In line with the above described loss of interneurons, ipsilaterally the frequency of spontaneous inhibitory post-synaptic currents (sIPSCs) before application and after washout of bicuculline was substantially lower compared to the contralateral side and sham controls (Figures 2C,D, left graphs). In contrast, sIPSC peak amplitudes were not different between groups, indicating unaltered post-synaptic receptor function (Figures 2C,D, right).

Collectively, these data indicate that, despite the loss of GABAergic interneurons and phasic inhibition, ambient GABA levels are preserved or even increased in the ipsilateral hippocampus.

### Reactive Astrocytes in the Sclerotic Hippocampus Display Pronounced GABA Accumulation

**Figure 3:**
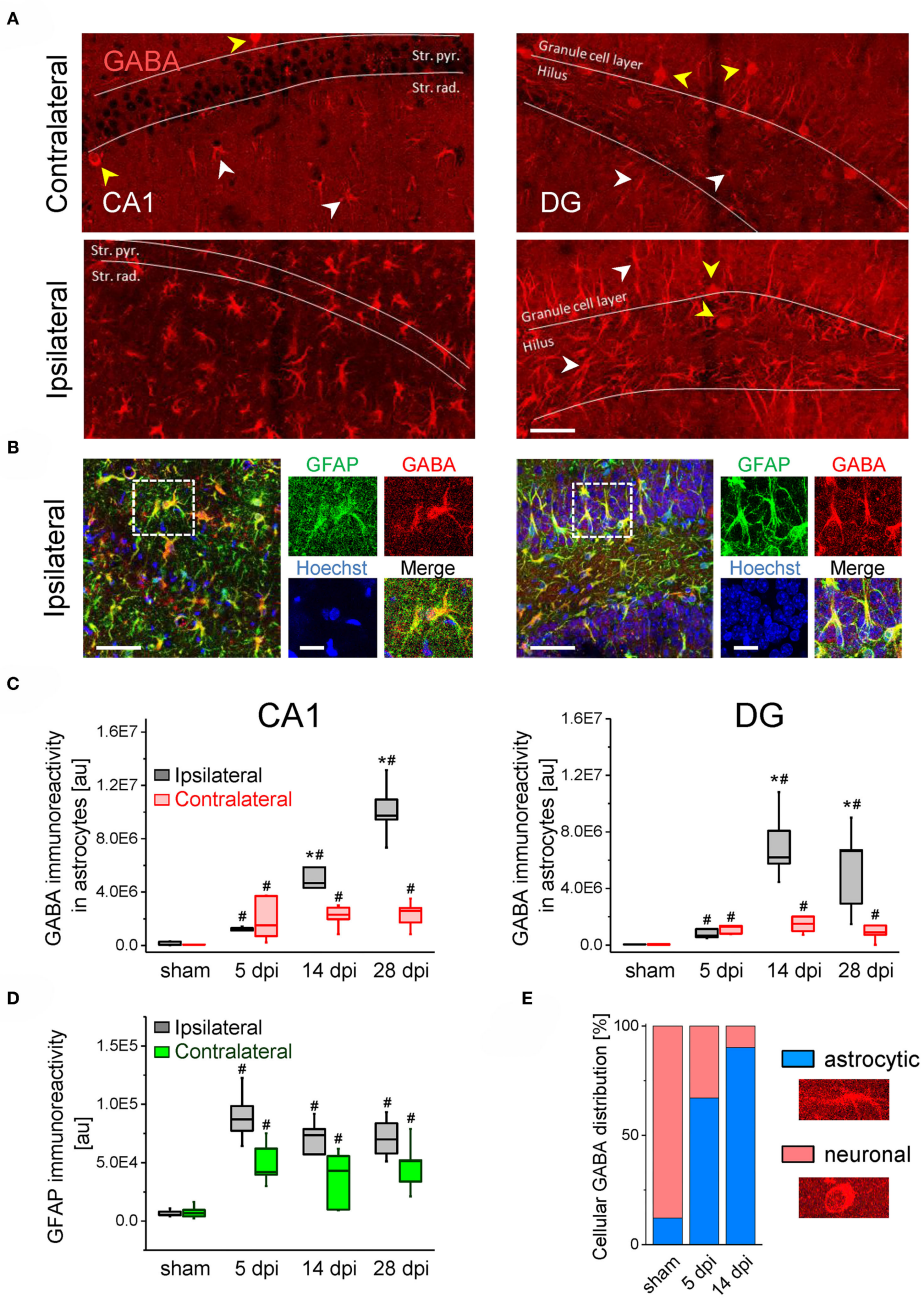
GABA immunostaining in the ispi- and contralateral hippocampus of kainate injected animals. **(A)** Representative confocal images of GABA staining in the hippocampal CA1 region (left panels) and the dentate gyrus (DG, right). Scale bar = 50 μm, white and yellow arrowheads denote GABA immunoreactivity in astrocytes and neurons, respectively. **(B)** GABA (red), GFAP (green) and Hoechst (blue) triple staining in the ipsilateral CA1 region (left panels) and DG (right). Dashed boxes in the left panels indicate areas enlarged to the right. Scale bars = 50 μm (large panels) and 20 μm (blowups). **(C)** Quantifications of GABA immunoreactivity in GFAP-positive astrocytes in the CA1 region and dentate gyrus at different time points following kainate or sham injection. **(D)** Quantifications of GFAP immunoreactivity in the CA1 region. **(E)** Relative cellular distribution of ipsilateral GABA immunoreactivity in the DG. N = 5 slices from five animals for each condition and time point. Error bars represent SD. *significantly different from the contralateral side, #significantly different from sham (*p* < 0.05, stratified two-way ANOVA followed by Tukey's test and independent samples *t*-test per group).

The preserved or aberrantly increased tonic GABA_A_R currents raised the question of the cellular origin of the transmitter. A number of studies have proposed that GABA produced and released by astrocytes significantly contributes to extrasynaptic GABA levels and tonic inhibition, especially under pathological conditions (22, 23, 32–34). To explore whether astrocytic GABA could account for the maintained/increased tonic currents in our TLE model, we performed immunostaining with antibodies against GABA and GFAP in hippocampal slices at different time points after kainate (5, 14, and 28 dpi) or sham injection (14 dpi). In the latter, strong GABA immunoreactivity was mainly seen in neurons while astrocytes were only weakly immunoreactive. Remarkably, in the ipsilateral hippocampus of kainate-treated mice, a strong increase in astrocytic GABA levels (~8-fold in the CA1 region and ~14-fold in the DG) was observed already 5 dpi, while maximal accumulation was reached 28 dpi in CA1 (~70-fold increase) and 14 dpi in the DG (~55-fold increase; Figures 3A–C). Contralaterally, astrocytic GABA was also elevated in both hippocampal regions and at all investigated time points, but compared to the ipsilateral side the increase at the later time points was significantly less (Figures 3A–C). GFAP immunoreactivity (the increase of which reflecting astrogliosis) showed a strong elevation already 5 dpi on both sides, which, however, did not increase further during the next 4 weeks (Figure 3D). We found a significant positive correlation between the astrocytic GABA content and GFAP immunoreactivity with a correlation factor of *r* = 0.65 (*p* < 0.000001).

In the DG of sham injected animals, 88% of total (astrocytic + neuronal) GABA was found in neurons and merely 12% in astrocytes. Intriguingly, after kainate injection the astrocytic contribution increased to 67% at 5 dpi and reached 90% at 14 dpi (Figure 3E). In the CA1 area astrocytic GABA increased from 38% in sham mice to 97% at 5 dpi and 99.5% at 14 dpi in kainate injected mice (not shown).

These data show that during epileptogenesis the loss of GABAergic neurons goes along with a pronounced increase in astrocytic GABA content. Release of GABA from astrocytes might thus mediate tonic inhibition in the epileptic hippocampus.

### Astrocytic GABA Synthesis Rather Than Uptake From the Extracellular Space Accounts for GABA Accumulation

**Figure 4:**
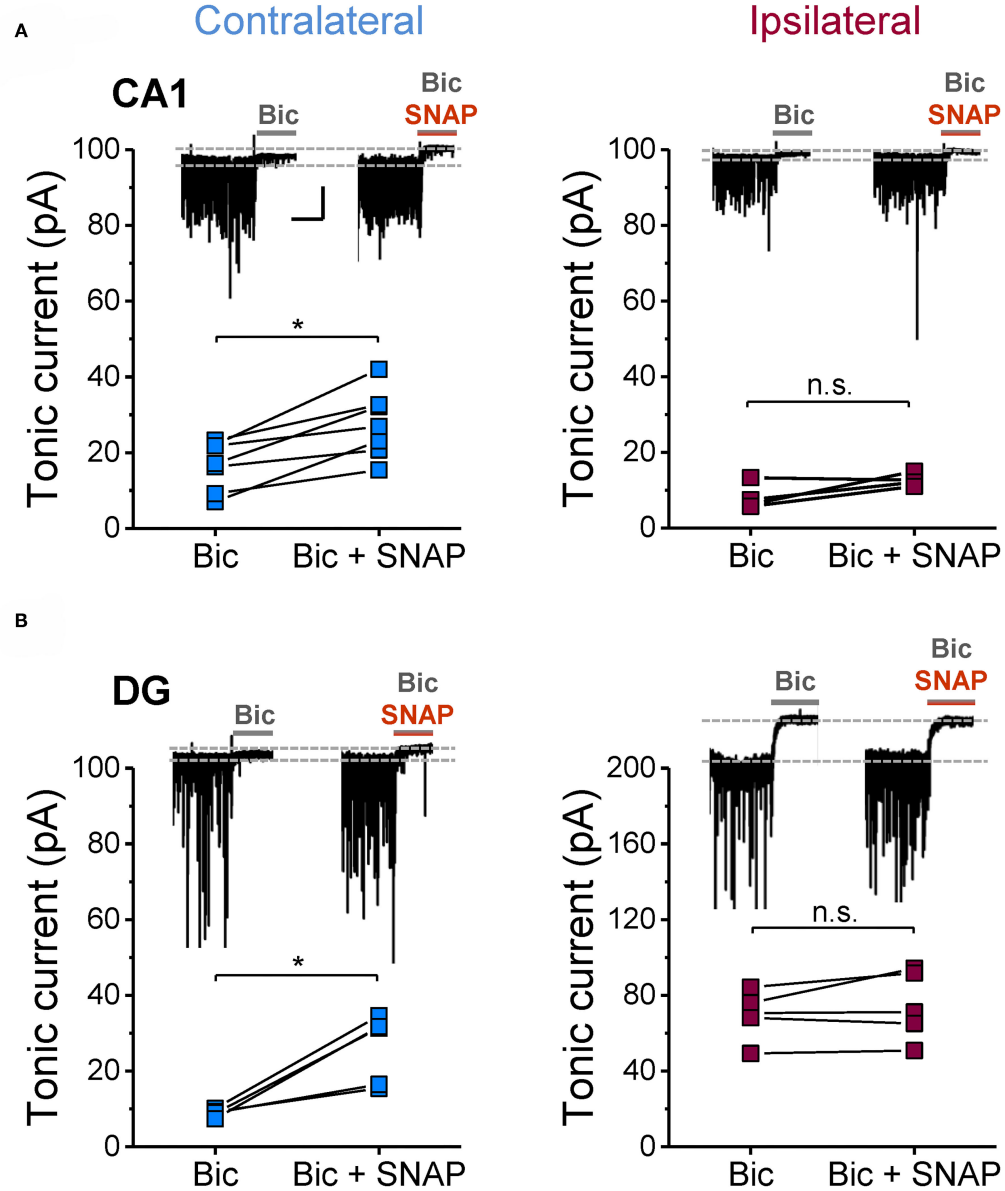
Effect of GAT3 inhibition on tonic inhibitory currents in the hippocampus of epileptic mice. The glial GABA transporter was blocked with SNAP-5114 (100 μM) and consequences on tonic inhibitory currents were tested in ipsi- and contralateral **(A)** CA1 neurons and **(B)** DG granule cells (14 dpi). Inhibition of GAT3 caused an increase in tonic currents on the contralateral but not on the ipsilateral side. Each data set was obtained from at least three mice. Calibration bars for original traces in **(A)** indicate 20 s and 50 pA and also apply to **(B)**. *significantly different (*p* < 0.05; paired *t*-test).

**Figure 5:**
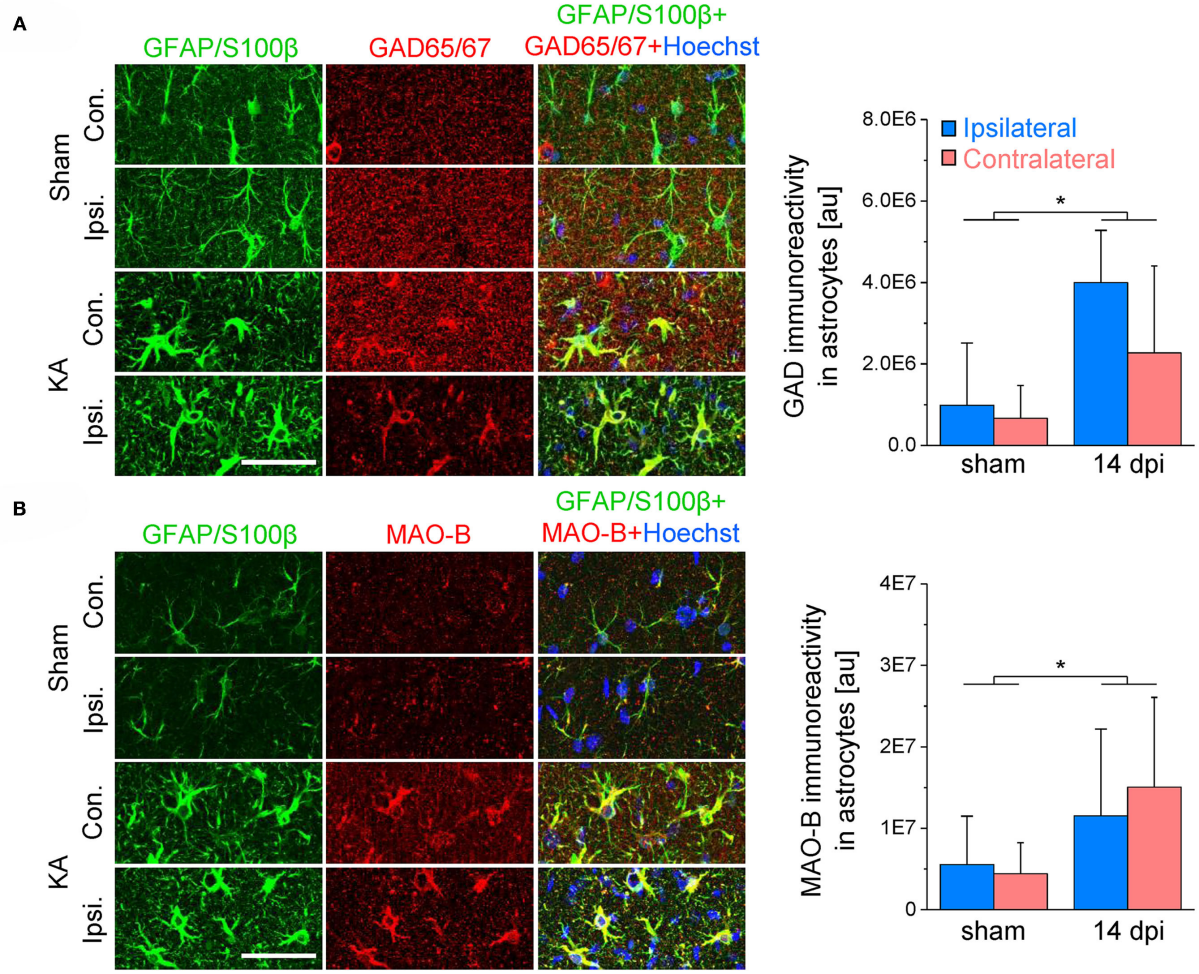
GAD65/67 and MAO-B immunoreactivity in the hippocampal CA1 region 2 weeks after epilepsy induction. **(A)** GAD65/67 (red), GFAP/S100β (green) and Hoechst (blue) staining in the ipsi- and contralateral CA1 region of sham and kainate injected animals. The graph (right) shows the quantification of GAD immunoreactivity in astrocytes. **(B)** MAO-B (red), GFAP/S100β (green) and Hoechst (blue) staining and quantification. *N* = 10 slices from five mice for each condition. Error bars represent SD. *significantly different (*p* < 0.05, two-way ANOVA). Scale bar = 50 μm.

Next we investigated potential mechanisms that might underlie astrocytic GABA accumulation. Astrocytes can acquire GABA in different ways: by uptake, reduced degradation or synthesis. To evaluate these potential mechanisms, we blocked glial GABA uptake with the GAT-2/3-specific inhibitor SNAP-5114 and utilized neuronal tonic GABA_A_R current as an indirect read-out of extracellular GABA levels. On the contralateral side, the blocker caused the expected increase in tonic current amplitudes in both CA1 and DG (65 and 191% increase, respectively). Ipsilaterally, however, SNAP-5114 had no effect (Figure 4), indicating lack of GABA transporter activity. This observation led us to conclude that astrocytic GABA accumulation in the ipsilateral hippocampus is not simply mediated by enhanced uptake from the extracellular space. Since SNAP-5114 had no effect, it is also unlikely that GABA was released from astrocytes through a reversed operation of the glial transporter (23, 34, 35). Astrocytes can synthesize GABA via decarboxylation of glutamate by glutamate decarboxylase (GAD) or through degradation of putrescine mediated primarily by monoamine oxidase B (MAO-B) (33, 36). We performed immunohistochemical analysis using antibodies against MAO-B and two isoforms of GAD, GAD67 and GAD65, to gain information about the expression levels of these enzymes in astrocytes. We have limited this study to the CA1 region because in human and experimental TLE GAD is strongly up-regulated in DG granule cells and mossy fibers, which complicates analysis in this region. In our TLE model, GAD and MAO-B immunoreactivity was significantly increased in GFAP/S100β-positive astrocytes of kainate injected mice as compared to sham injected controls. However, there was no difference in GAD- or MAO-B-immunoreactivity between the ipsi- and contralateral hippocampus of kainate injected mice (Figure 5).

Taken together, these results indicate that astrocyte GABA accumulation in epilepsy is mediated by glutamate decarboxylation and monoacetylation of putrescine but not by uptake from the extracellular space.

## Discussion

**Figure 6:**
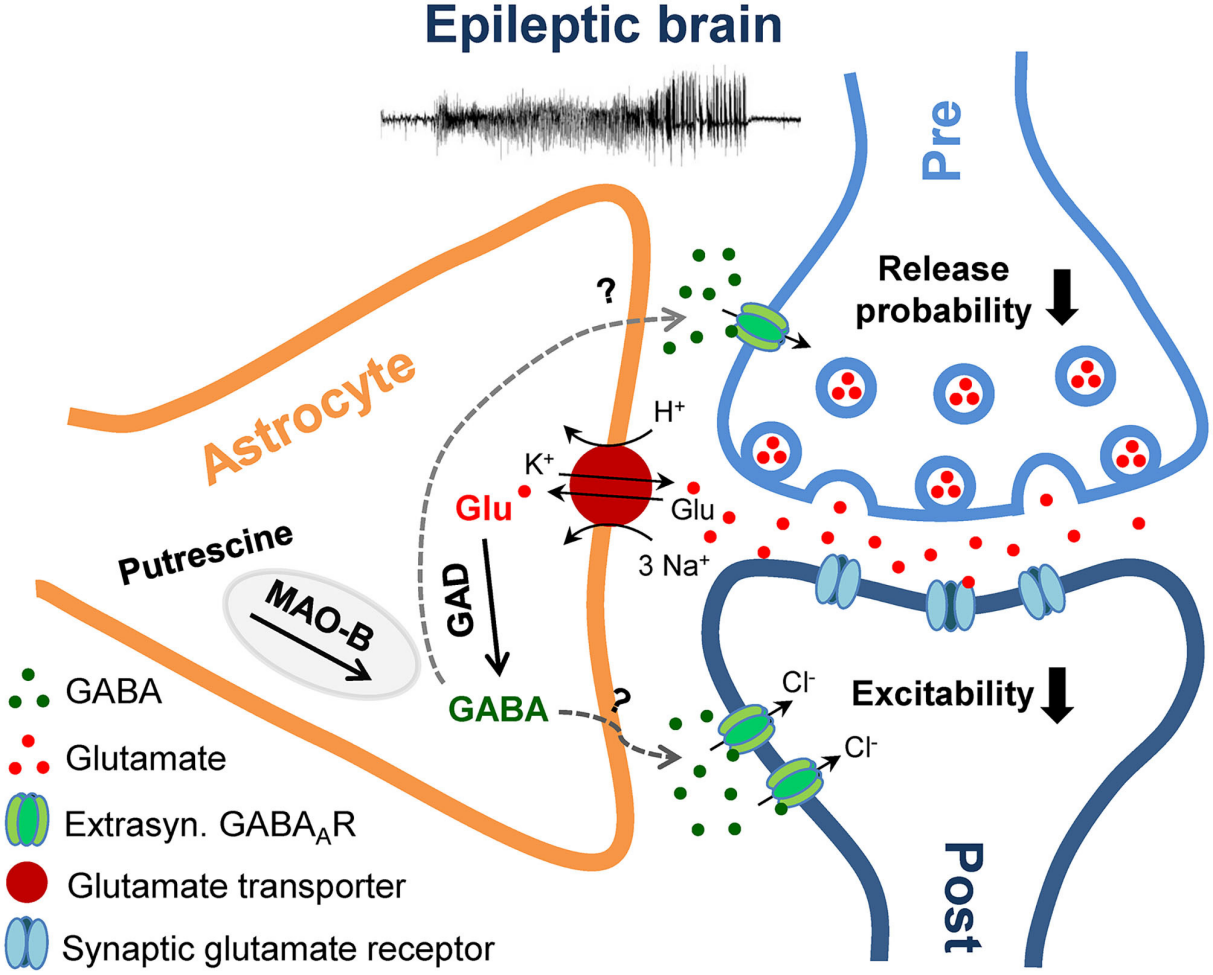
Schematic illustration of the proposed mechanism and role of astrocytic GABA overproduction in the epileptic brain. Epileptic activity triggers astrocytic GABA production via decarboxylation of glutamate (Glu) by glutamate decarboxylase (GAD) and degradation of putrescine by monoamine oxidase B (MAO-B). Upon release into the extracellular space via a yet unknown mechanism, GABA activates high affinity extrasynaptic GABA_A_ receptors (GABA_A_R) on excitatory neurons and elicits a tonic inhibitory Cl^−^ current, which inhibits synaptic transmission and neuronal excitability.

In the present study we examined the hypothesis that in the sclerotic epileptic hippocampus, increased GABA release from reactive astrocytes counterbalances the reduced neuronal release, caused by loss of interneurons, resulting in preserved tonic inhibition. Two weeks after epilepsy induction in our model, ipsilaterally we observed severe interneuronal loss, preserved or elevated tonic inhibitory currents in CA1 pyramidal neurons or dentate granule cells and a pronounced increase in astrocytic GABA immunoreactivity. Together with the lack of GABA transporter activity and increased GAD65/67 and MAO-B expression, the most plausible interpretation of our results is that during excessive neuronal activity astrocytes overproduce GABA through *de novo* synthesis and decarboxylation of excess glutamate, which after release into the extracellular space activates tonic GABA_A_R-mediated currents in excitatory neurons and reduces their excitability (Figure 6). In fact, in view of the dramatic loss of interneurons, the question is not why these mice have seizures, but rather why they are seizure-free most of the time. The observed GABA accumulation in reactive astrocytes might reflect a compensatory mechanism aimed to restore excitation-inhibition balance in TLE. However, as speculated earlier (18), the compensation probably generates a less stable network that fails whenever an epileptic seizure occurs. On the other hand, several reports suggested that due to altered expression of the Cl^−^ transporters NKCC1 and KCC2 (and therefore altered neuronal Cl^−^ homeostasis), GABA may have excitatory effects in epilepsy (37–40). If true, astrocytic GABA would not counteract but exacerbate seizures. This scenario is, however, unlikely for several reasons. First, using immunohistochemical analysis we did not observe KCC2 down-regulation in our model (data not shown). Second, in a recent study Pandit et al. (21) suggested that reactive astrocytes release GABA through Bestrophin 1 (Best1) anion channels. Consistent with an anti-epileptic function of astrocytic GABA, in two different TLE models, mice with Best 1 deletion displayed increased seizure susceptibility, an effect that could be reversed by astrocyte-specific re-expression of Best1. Finally, it must be stressed that loss of inhibition with a concomitant excitatory effect of astrocytic GABA would make the occurrence of seizure-free periods difficult to explain. Hence, the most reasonable conclusion that can be drawn from our results is that excessive astrocytic GABA production and release represents a compensatory mechanism in epilepsy.

### Loss of Interneurons but Preserved or Increased Tonic Inhibition in the Sclerotic Hippocampus

Loss of interneurons and the consequential impairment of GABAergic inhibition has been regarded as the main cause of seizure activity (16). However, there is still controversy about the extent of the loss and the relative vulnerability of different interneuron subtypes in human epilepsy (5–9). Already at 5 dpi, our immunohistochemical analysis revealed a pronounced loss of PARV-positive and GABA-positive interneurons in the sclerotic CA1 region, and a strong reduction in the ipsilateral dentate gyrus. This early timing and massive extent of cell loss in our intra*cortical* model agrees with data from the intra*hippocampal* kainate TLE model where a similar decline in interneuron numbers was evident already 1–2 dpi (12, 13). *In situ* hybridization of GAD67 mRNA (12) or staining against the α1 subunit of the GABA_A_R (13) demonstrated that the loss was attributable to cell death and not merely down-regulation of PARV, as suggested previously (41–43). Our GABA staining together with the observed reduction in sIPSC frequency (reflecting reduced synaptic GABA release) supports this view.

Previous work has suggested that GABA spillover from synapses represents the main source of ambient GABA under physiological conditions (31), and thus one would have expected decreased tonic currents after interneuronal loss. However, several studies reported preserved or even increased tonic inhibition in experimental and human TLE (18, 44–50). Consistent with these studies, 14 dpi we detected strongly increased or preserved tonic inhibitory currents in ipsilateral dentate granule and CA1 pyramidal cells, respectively. Pandit and colleagues also reported preserved tonic GABA currents in CA1 neurons 15 days after intracerebroventricular kainate injection, which was preceded by a transient increase at 3 dpi (21). Although this time point was not evaluated in the present study, it is tempting to speculate that an early increase in tonic inhibition might contribute to the suppression of seizures during the latent period in our model.

### Astrocytic GABA Production and Release

The preserved/increased tonic GABA currents in epilepsy might be explained either by GABA_A_R plasticity or preserved/increased extracellular GABA concentrations (18). Concerning the latter, it has been argued that reactive astrocytes are the main source of ambient GABA in epilepsy (21, 31). In agreement with this view, we detected massive GABA accumulation in reactive astrocytes already 5 dpi in both, the CA1 region and the dentate gyrus. Accumulation was even more pronounced 14 and 28 dpi and positively correlated with GFAP immunoreactivity. Indeed, GABA accumulation in reactive astrocytes has been observed in different brain pathologies, including Alzheimer's disease, Parkinson's disease, stroke and epilepsy (22–25), suggesting that it represents a general feature of the astrocytic reaction. Increasing evidence suggests that astrocyte changes associated with astrogliosis play a detrimental role in epileptogenesis (51–53), a view that is not compatible with an assumed protective effect of astrocytic GABA release. On the other hand, reactive astrocytes might play a dual role in CNS pathologies (54), and whether they exert pro- or antiepileptic effects in epilepsy probably depends on a number of factors, including etiology, timing and severity of epilepsy as well as environmental conditions and interactions with other factors (e.g., inflammatory mediators).

Interestingly, astrocytic GABA was also enhanced contralaterally. Given the absence of interneuron degeneration, it is somewhat surprising that tonic inhibition was not affected on this side. Differences in the extent of GABA increase or, more likely, in astrocytic GABA release might account for this phenomenon. Ictal activity may trigger GABA production on the contralateral side (as indicated by MAO-B and GAD up-regulation), but not affect expression and/or activation of the release machinery. It is also conceivable that astrocytic GABA release occurs only in the absence of GABA uptake. Clearly, the functional significance of astrocytic GABA on the contralateral side remains to be established and requires further studies.

Since the latent period of epileptogenesis in our model lasts about 5 days (27), the increased GABA immunoreactivity detected at the three different time points indicates that astrocyte GABA release influences both development and progression of TLE. The time course of GABA accumulation in our model differs from that reported recently for intracerebroventricular injection where astrocytic GABA peaked 3 dpi and then returned to control level (21). This transient increase implies a role for astrocytic GABA release in inception, rather than progression, of TLE. Differences between models might account for this discrepancy. Indeed, we injected 300 ng kainate into the neocortex just above the right dorsal hippocampus, while Pandit and colleagues applied 100 ng of the drug into the ventricle. In our model, initiation of epileptogenesis should therefore be faster and more focal. Our data are not in line with the concept that GABA was released through reversed operation of astrocytic GABA transporters, as suggested earlier (23, 34). Recent studies provided evidence that reactive astrocytes in epilepsy and other CNS pathologies release GABA through Best1 anion channels (21, 22, 24). Whether this mechanism underlies GABA release in our model remains to be investigated. Our immunohistochemical analysis showed increased immunoreactivity for both GAD and MAO-B, indicating that astrocytic GABA synthesis occurs through multiple routes. Involvement of GAD in the process is at odds with studies reporting that astrocytic GABA production is primarily accomplished by putrescine degradation via MAO-B (22, 24, 25, 33, 55). However, decarboxylation of glutamate via astrocytic GAD may represent an epilepsy-specific mechanism, triggered by the excessive astrocytic glutamate uptake during neuronal hyperactivity. Which of the GAD isoforms (GAD65 or GAD67) is expressed and/or up-regulated in reactive astrocytes cannot be deduced from our analysis. Previous studies have shown that astrocytes in the healthy brain express GAD67 but not GAD65 (56). Although this suggests that overproduction of astrocytic GABA in TLE is mediated by GAD67, a participation of GAD65 cannot be excluded.

## Conclusion

In this study we show that despite massive interneuron loss, tonic GABA_A_R currents are preserved in CA1 pyramidal neurons and increased in dentate granule cells of the sclerotic hippocampus in a chronic TLE model. Furthermore, we gained evidence that GABA overproduction and release from reactive astrocytes represents the main source of ambient GABA responsible for inhibition under this condition. As human and rodent astrocytes display many similar functional properties (57), it is reasonable to assume that this form of inhibition is also involved in genesis and/or progression of human TLE. Hence, molecules that stimulate or improve astrocyte GABA production or release might have effective antiepileptogenic properties.

## Data Availability Statement

The raw data supporting the conclusions of this article will be made available by the authors, without undue reservation.

## Ethics Statement

The animal study was reviewed and approved by LANUV 84-02.04.2012.A212, 84-02-04.2015.A393.

## Author Contributions

JM designed experiments, acquired, analyzed, and interpreted data. AT and LH acquired, analyzed, and interpreted data. HM analyzed and interpreted data. CS and PB designed and supervised experiments and wrote the manuscript. All authors revised the work critically and approved the manuscript.

## Conflict of Interest

The authors declare that the research was conducted in the absence of any commercial or financial relationships that could be construed as a potential conflict of interest.
